# Sex Differences in the Factors Associated With Lifetime Alcohol, Tobacco and Cannabis Use Among Ghanaian University Students: A Cross‐Sectional Study

**DOI:** 10.1002/puh2.70250

**Published:** 2026-04-27

**Authors:** Yula Salifu, Joseph Lasong, Amidu Alhassan, Frank Offei Odonkor, Torjim Salifu

**Affiliations:** ^1^ Department of Population Family and Reproductive Health School of Public Health University of Ghana, Legon Accra Ghana; ^2^ Department of Population and Reproductive Health School of Public Health University for Development Studies Tamale Ghana; ^3^ Department of Adult Health School of Nursing and Midwifery University of Cape Coast Cape Coast Ghana

**Keywords:** alcohol, cannabis, Ghana, lifetime substance use, prevalence rate ratio, sex differences, sub‐Saharan Africa, tobacco, university students

## Abstract

**Background:**

Substance use among university students in Ghana is a pressing concern, with pronounced sex differences yet poorly characterised determinants. This study examined factors associated with lifetime alcohol, tobacco and cannabis use among undergraduate students, with explicit attention to sex‐specific patterns.

**Methods:**

A cross‐sectional study was conducted from January to March 2023 at the University for Development Studies, Tamale. A total of 600 undergraduate students were recruited using quota sampling proportional to school, year and sex, followed by simple random selection (response rate 100%). Lifetime use was defined as self‐reported use of each substance at least once, measured using an adapted World Health Organization (WHO) Student Drug Use Survey. Sex‐stratified modified Poisson regression with robust standard errors estimated prevalence rate ratios (aPRRs) and 95% confidence intervals (CIs).

**Results:**

Lifetime prevalence was 17.7% for alcohol, 17.0% for tobacco and 16.7% for cannabis. Males reported higher use across all substances (alcohol: 31.1%; tobacco: 30.6%; cannabis: 27.4%) than females (11.3%, 10.6%, 11.6%). In the total sample, male sex (aPRR 2.01), older age (>24 years) (1.85) and peer or family drug history (1.41–1.48) predicted higher alcohol use, whereas Islamic faith (0.59) and residing at a friend's house (0.49) were protective. Male sex (3.12) and non‐Christian religion (3.28) predicted tobacco use. Male sex (2.56) was the only pooled predictor of cannabis use. Sex‐stratified analyses revealed distinct patterns: strict parental supervision and absence of family conflict protected males against alcohol use; Islamic faith protected females; residential autonomy and persistent family conflict were female‐specific cannabis risk factors.

**Conclusions:**

Sex differences profoundly shape substance use among university students in Ghana. Religion, parental oversight and family dynamics are key protective or risk factors that differ by sex. Prevention strategies must be sex‐sensitive and culturally grounded.

## Graphical abstract

Among 600 Ghanaian university students, males had higher lifetime alcohol, tobacco, and cannabis use than females. Strict parental supervision protected males; Islamic faith protected females; residential autonomy and family conflict increased female cannabis use. Prevention strategies must be sex‐sensitive and culturally grounded.

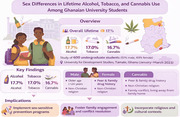

## 1 Introduction

Globally, alcohol remains the most frequently consumed psychoactive substance among young adults. The 2024 World Health Organization (WHO) Global Status Report on Alcohol and Health estimates past‐year alcohol use among adolescents aged 15–19 years at 22.5% globally, though prevalence increases substantially in the 18–29‐year age group across most world regions [1]. However, these global averages mask substantial regional variation, with sub‐Saharan African countries recording considerably higher rates. A recent systematic review and meta‐analysis reported a pooled lifetime prevalence of alcohol use among young people in sub‐Saharan Africa of 36.2%, and a pooled 12‐month prevalence of 30.0% [2]. Furthermore, a study among university students in South Africa found that 80.6% reported alcohol use, whereas 46% reported cannabis use [3]. These figures underscore the magnitude of the problem across the region.

University environments, characterised by increased social autonomy, academic pressure and limited parental oversight, are particularly conducive to substance use experimentation. Gebreslassie et al. [4] reported that 34.5% of university students in Ethiopia had used alcohol at least once, with 9.5% reporting cigarette use and 28.7% reporting khat use. Globally, common risk factors include peer influence, academic stress, urban residence and proximity to drug sources on or near campus [5]. A comparative study by Stempliuk et al. [6] in Brazil demonstrated significant gender disparities, with male students reporting substantially higher rates of tobacco and cannabis use than females, a pattern consistent with literature from multiple African settings.

In Ghana, substance use among university students represents a significant and growing public health burden. A lifetime alcohol use prevalence of 54.8% has been documented among tertiary students in Ghana [7]. Benzodiazepine and cannabis use have also been reported, albeit at lower rates. Substance use in this context is frequently accompanied by psychological distress, academic disengagement and risky sexual behaviour [8]. Protective factors identified in the Ghanaian context include residing in university hostels, frequent religious engagement and strong familial bonds [9]. Nevertheless, poor coping mechanisms, ready access to substances and limited campus‐level interventions continue to leave many students vulnerable.

Sex‐based differences in substance use patterns are well‐established among Ghanaian university students, with males consistently reporting higher rates of alcohol, tobacco and cannabis use than females [10]. These disparities reflect a complex interplay of social, cultural and psychological forces. In many African societies—including Ghana—substance use, particularly alcohol and tobacco, is more socially sanctioned among males, contributing to higher rates of initiation and sustained use [8]. Peer influence is especially salient for male students, who are more likely to inhabit social environments that normalise substance use during social gatherings and recreational activities [11, 12]. Moreover, gender‐based differences in coping strategies have been documented: Male students are more likely to use psychoactive substances as a mechanism for coping with academic stress, anxiety or emotional discomfort, whereas female students tend to rely on social support networks and religious engagement [7]. Female students may also face stronger familial and social restrictions that deter substance use, notwithstanding their exposure to comparable stressors [12, 13]. These gendered dynamics highlight the need for sex‐specific approaches in prevention research and practice.

Despite a growing body of research on substance use among young people globally and in sub‐Saharan Africa [2, 10, 13, 14, 15, 16, 17], sex‐specific determinants of the lifetime use of alcohol, tobacco and cannabis among university students in Ghana remain insufficiently characterised. Prior studies have predominantly reported aggregate prevalence estimates and general risk factors [10, 18, 19, 20], with limited attention to sex‐stratified analysis. This gap hinders the development of gender‐responsive, evidence‐based prevention strategies tailored to higher education settings. The present study is part of a wider survey of substance use conducted at University for Development Studies (UDS), Ghana, for which the methods and some broader findings have been previously described [21]. This study therefore specifically examines sex differences in factors associated with lifetime alcohol, tobacco and cannabis use among undergraduate students at UDS, Tamale, Ghana.

## 2 Materials and Methods

### 2.1 Study Design, Setting and Period

A school‐based cross‐sectional study was conducted between January and March 2023 at the UDS, Tamale, in the Northern Region of Ghana. UDS is the first public university established in Northern Ghana and serves over 10,000 enrolled undergraduates across health‐based and non‐health‐based schools, faculties and departments. The cross‐sectional design was appropriate for estimating the lifetime prevalence of substance use and identifying associated factors at a defined point in time. However, it precludes causal inference; all reported associations should therefore be interpreted as cross‐sectional.

### 2.2 Study Population, Inclusion and Exclusion Criteria

The study targeted all registered undergraduate students at UDS. Students present on campus during the data collection period who voluntarily provided informed consent (or assent plus parental/guardian consent for those under 18 years) were eligible for inclusion. Students who declined participation, those who were severely ill during data collection, and those who returned incomplete questionnaires were excluded.

### 2.3 Sample Size and Sampling Procedure

The minimum required sample size was estimated using the Cochran formula: *N* ≥ *Z* ^2^ *pq*/*d* ^2^, where *Z* = 1.96 (95% confidence level), *p* = 0.899 (lifetime substance use prevalence from a comparable Cameroonian university study [22]), *q* = 1 − *p* and *d* = 0.05 (margin of error). Incorporating a 10% non‐response allowance yielded a minimum of 145; however, a final target of 600 was adopted to ensure adequate statistical power for sex‐stratified sub‐group analyses.

Sampling was carried out using a two‐stage process. First, quota sampling proportional to school, faculty, department, class year and sex distribution was applied to allocate the target sample, ensuring all structural units of the university were represented. Second, within each quota cell, individual students were selected by simple random sampling from departmental enrolment and registration records. The overall response rate was 100% (600/600): all recruited students who met the eligibility criteria completed and returned the electronic questionnaire within the stipulated 2‐week submission window. The 600 reported represents the final analytic sample. The sex distribution of the sample (67.8% female, 32.2% male) reflects the actual sex composition of the UDS student population during the study period and not unequal sampling effort. A detailed account of the sampling procedure for the wider study is reported in Lasong et al. [21].

### 2.4 Data Collection, Instrument and Quality Assurance

Data collection was guided by an adapted version of the WHO Student Drug Use Survey instrument [23], a standardised and internationally validated framework for measuring substance use in educational settings. In addition to this validated base instrument, the questionnaire was further refined through (i) a review of relevant literature [21, 24, 25] and (ii) a content validity assessment conducted by an expert review panel comprising three researchers with specialised expertise in substance use epidemiology and public health survey methodology, who evaluated each item for conceptual accuracy, contextual relevance and cultural appropriateness for Northern Ghanaian university students.

On the basis of the expert panel's feedback, question wording was modified for cultural clarity, response option categories were adjusted to reflect locally relevant substance use patterns, and the ordering of items was revised to minimise response fatigue. The revised questionnaire was pre‐tested among 30 undergraduate students at a neighbouring higher education institution. Pre‐test feedback led to rewording of three ambiguous items, simplification of two instruction sections and the removal of one item that duplicated information captured elsewhere.

The final questionnaire captured sociodemographic characteristics (sex, age, year of study, religion, marital status, residence status) and psychosocial determinants (parental co‐residence during upbringing, parental/guardian supervision style, frequency of family conflict and whether relatives or peers used drugs during the respondent's upbringing). In this article, substance use was limited to (i) lifetime alcohol consumption; (ii) lifetime tobacco smoking and (iii) lifetime cannabis smoking. The operational definition of ‘lifetime use’ applied throughout was self‐reported use of a given substance at least once in the respondent's lifetime, consistent with standard WHO survey definitions [23]. All substance use data were self‐reported.

The questionnaire was administered electronically via an anonymous link distributed through students’ WhatsApp and email accounts. Students were given 2 weeks to complete and submit their responses; reminders were issued every 2 days via text message, phone call and email. Participants below 18 years provided written assent alongside their parents’ or guardians’ written informed consent before inclusion; those aged 18 years and above provided written informed consent independently. Data were anonymised, and only the principal investigator retained access to the identifiable data linking file. Completed questionnaires were reviewed daily for completeness.

### 2.5 Statistical Analysis

Data were extracted, checked and cleaned in Microsoft Excel before being exported to STATA/MP 17.0 for analysis. Frequencies and proportions were computed for all categorical variables. Sex differences in participant characteristics and substance use outcomes were assessed using Pearson's chi‐square test of independence for all categorical comparisons; Fisher's exact test was applied for cells with an expected count below five. The *p* values in Table 1 reflect these sex‐by‐category comparisons (male vs. female). No post hoc pairwise comparisons were performed because all comparisons were binary (male vs. female). Statistical significance was defined as *p* < 0.05 throughout.

**TABLE 1:** Participant characteristics stratified by sex (*N* = 600).

Variables	Female (*n* [%])	Male (*n* [%])	*χ* ^2^, *p* value	Total (*n* [%])
Sex				
Female	—	—		407 (67.8)
Male	—	—		193 (32.2)
Age group (years)			14.77, *p* < 0.001^***^	
≤24	330 (81.1)	129 (66.8)		459 (76.5)
>24	77 (18.9)	64 (33.2)		141 (23.5)
Religion			9.23, *p* = 0.010^*^	
Christianity	209 (51.4)	90 (46.6)		299 (49.8)
Islam	196 (48.2)	96 (49.8)		292 (48.7)
Other(s)	2 (0.5)	7 (3.6)		9 (1.5)
Marital status			4.29, *p* = 0.117	
Married/Cohabiting	48 (11.8)	18 (9.3)		66 (11.0)
Single/Never married	359 (88.2)	175 (90.7)		534 (89.0)
Residence status			4.53, *p* = 0.104	
Family house	158 (38.9)	90 (46.6)		248 (41.3)
Renting/Living alone	227 (55.9)	98 (50.8)		325 (54.2)
Friend's house	21 (5.2)	5 (2.6)		26 (4.3)
Year of study			9.30, *p* = 0.098	
Year 1	100 (24.6)	32 (16.6)		132 (22.0)
Year 2	71 (17.4)	33 (17.1)		104 (17.3)
Year 3	79 (19.4)	31 (16.1)		110 (18.3)
Year 4	64 (15.7)	40 (20.7)		104 (17.3)
Year 5	31 (7.6)	22 (11.4)		53 (8.8)
Year 6	62 (15.3)	35 (18.1)		97 (16.2)
Parents together when growing up			38.33, *p* < 0.001^***^	
No	59 (14.5)	71 (36.8)		130 (21.7)
Yes	348 (85.5)	122 (63.2)		470 (78.3)
Parental/Guardian supervision			25.90, *p* < 0.001^***^	
Liberal	34 (8.4)	32 (16.6)		66 (11.0)
Strict	250 (61.4)	77 (39.9)		327 (54.5)
Moderate	123 (30.2)	84 (43.5)		207 (34.5)
Family quarrels			1.32, *p* = 0.517	
Sometimes	165 (40.5)	87 (45.1)		252 (42.0)
Always	94 (23.1)	44 (22.8)		138 (23.0)
Never	148 (36.4)	62 (32.1)		210 (35.0)
Relatives using drugs when growing up			1.72, *p* = 0.189	
No	232 (57.0)	99 (51.3)		331 (55.2)
Yes	175 (43.0)	94 (48.7)		269 (44.8)
Friends using drugs when growing up			0.09, *p* = 0.760	
No	252 (61.9)	122 (63.2)		374 (62.3)
Yes	155 (38.1)	71 (36.8)		226 (37.7)
Lifetime alcohol use			35.24, *p* < 0.001^***^	
No	361 (88.7)	133 (68.9)		494 (82.3)
Yes	46 (11.3)	60 (31.1)		106 (17.7)
Lifetime tobacco use			37.13, *p* < 0.001^***^	
No	364 (89.4)	134 (69.4)		498 (83.0)
Yes	43 (10.6)	59 (30.6)		102 (17.0)
Lifetime cannabis use			23.87, *p* < 0.001^***^	
No	360 (88.4)	140 (72.5)		500 (83.3)
Yes	47 (11.6)	53 (27.4)		100 (16.7)

*Note: p* values are from Pearson's chi‐square test of independence (or Fisher's exact test where expected cell counts <5) comparing male and female distributions. Liberal: minimal parental oversight; strict: close, regular monitoring of activities; moderate: balanced supervision. Sometimes: occasional family conflict; always: persistent/frequent family conflict; never: no family conflict. Other(s): any religious affiliation other than Christianity or Islam.

^***^ *p* < 0.001.

^*^ *p* < 0.05.

Sex‐stratified multivariable analyses were conducted separately for three strata: the total sample, males only and females only. For each stratum and each outcome (lifetime alcohol use, lifetime tobacco use and lifetime cannabis use), an adjusted modified Poisson regression model with a log link and robust standard errors was fitted [26]. All candidate predictor variables—selected a priori based on clinical and epidemiological plausibility—were entered simultaneously into each model (fully adjusted specification). The choice of modified Poisson regression, rather than logistic regression, was guided by the observation that all three outcomes had a prevalence exceeding 10%, a threshold beyond which the odds ratio from logistic regression systematically overestimates the prevalence ratio [26]. Robust standard errors were estimated using the *vce(robust)* command in STATA, enhancing the reliability of inference under potential model misspecification. Results are presented as adjusted prevalence rate ratios (aPRRs) with 95% confidence intervals (CIs) and corresponding significance levels.

One near‐zero aPRR estimate was produced for female participants residing at a friend's house in the alcohol model (aPRR ≈ 1.62 × 10^−7^). This extreme estimate reflects quasi‐complete separation—a recognised numerical phenomenon in maximum‐likelihood estimation that arises when no individual in a particular exposure category experienced the outcome (i.e., no female residing at a friend's house reported lifetime alcohol use). This estimate is retained in Table 2 for transparency but is flagged with a footnote and is not substantively interpreted.

**TABLE 2:** Adjusted prevalence rate ratios (aPRR) and 95% confidence intervals (CI) for lifetime alcohol use, stratified by sex.

Variables	Total aPRR (95% CI)	Male aPRR (95% CI)	Female aPRR (95% CI)
Sex			
Female (ref)	1.00 (ref)	—	—
Male	2.00 (1.39–2.90)^***^	—	—
Age (years)			
≤24 (ref)	1.00 (ref)	1.00 (ref)	1.00 (ref)
>24	1.85 (1.14–3.02)^*^	3.54 (1.62–7.74)^**^	0.96 (0.47–1.94)
Religion			
Christianity (ref)	1.00 (ref)	1.00 (ref)	1.00 (ref)
Islam	0.59 (0.41–0.84)^**^	0.63 (0.40–0.99)	0.51 (0.28–0.93)^*^
Other(s)	1.31 (0.72–2.37)	1.19 (0.39–3.61)	4.67 (0.72–30.11)
Marital status			
Married (ref)	1.00 (ref)	1.00 (ref)	1.00 (ref)
Single	1.11 (0.71–1.73)	0.90 (0.47–1.73)	2.44 (0.82–7.30)
Residence status			
Family house (ref)	1.00 (ref)	1.00 (ref)	1.00 (ref)
Renting/Alone	0.82 (0.54–1.26)	0.74 (0.39–1.39)	1.37 (0.76–2.45)
Friend's house	0.49 (0.25–0.97)^*^	1.27 (0.41–3.89)	≈1.62 × 10^−^^7^ ^[Link]^, ^[Link]^
Year of study			
Year 1 (ref)	1.00 (ref)	1.00 (ref)	1.00 (ref)
Year 2	0.87 (0.40–1.88)	1.07 (0.37–3.09)	0.59 (0.17–2.03)
Year 3	1.12 (0.53–2.38)	0.74 (0.25–2.19)	1.11 (0.40–3.08)
Year 4	1.36 (0.63–2.92)	0.67 (0.22–2.05)	1.65 (0.54–5.03)
Year 5	0.90 (0.40–2.03)	0.76 (0.24–2.42)	0.44 (0.10–1.97)
Year 6	1.46 (0.69–3.09)	1.07 (0.37–3.10)	1.02 (0.37–2.82)
Parents together (growing up)			
No (ref)	1.00 (ref)	1.00 (ref)	1.00 (ref)
Yes	1.36 (0.84–2.19)	2.65 (1.50–4.69)^*^	0.63 (0.26–1.54)
Parental supervision			
Liberal (ref)	1.00 (ref)	1.00 (ref)	1.00 (ref)
Strict	0.48 (0.27–0.86)	0.43 (0.22–0.83)^*^	0.56 (0.21–1.50)
Moderate	0.92 (0.55–1.55)	0.62 (0.31–1.26)	1.04 (0.45–2.41)
Family quarrels			
Sometimes (ref)	1.00 (ref)	1.00 (ref)	1.00 (ref)
Always	0.89 (0.62–1.27)	0.74 (0.46–1.19)	0.62 (0.29–1.33)
Never	0.52 (0.33–0.82)^**^	0.45 (0.24–0.83)^*^	0.51 (0.24–1.09)
Relatives using drugs (growing up)			
No (ref)	1.00 (ref)	1.00 (ref)	1.00 (ref)
Yes	1.41 (1.02–1.95)^*^	1.15 (0.78–1.70)	1.97 (1.09–3.55)^*^
Friends using drugs (growing up)			
No (ref)	1.00 (ref)	1.00 (ref)	1.00 (ref)
Yes	1.48 (1.04–2.10)^*^	1.21 (0.76–1.94)	1.68 (0.94–3.02)

*Note:* All models simultaneously adjusted for all listed variables.

Abbreviations: aPRR, adjusted prevalence rate ratio; CI, confidence interval; ref; reference category.

^a^Near‐zero estimate reflects quasi‐complete separation (no female in this residence category reported alcohol use); not substantively interpreted.

^***^ *p* < 0.001.

^**^ *p* < 0.01.

^*^ *p* < 0.05.

## 3 Results

### 3.1 Participant Characteristics

The final analytic sample comprised 600 undergraduate students (response rate: 100%), of whom 407 (67.8%) were female and 193 (32.2%) were male, reflecting the UDS student population sex composition. Most participants (76.5%) were aged 24 years or younger; 49.8% identified as Christian, 48.7% as Muslim, and 1.5% reported other religious affiliations. Nearly all participants (89.0%) were single or never married, and 54.2% resided in rented accommodation or lived independently. Participant characteristics stratified by sex are presented in Table 1.

Statistically significant sex differences were observed in age distribution (*χ* ^2^ = 14.77, *p* < 0.001), religion (*χ* ^2^ = 9.23, *p* = 0.010), parental co‐residence during upbringing (*χ* ^2^ = 38.33, *p* < 0.001) and nature of parental/guardian supervision (*χ* ^2^ = 25.90, *p* < 0.001). Males were more likely to be aged over 24 years (33.2% vs. 18.9%), to have grown up without both parents in the home (36.8% vs. 14.5%) and to have experienced liberal parental supervision (16.6% vs. 8.4%). Marital status, residence, year of study, family quarrels and histories of relatives or friends using drugs did not differ significantly by sex (all *p* > 0.05). Overall lifetime substance use prevalence was 17.7% for alcohol, 17.0% for tobacco and 16.7% for cannabis, with males reporting substantially higher rates across all three substances (alcohol: 31.1% vs. 11.3%; tobacco: 30.6% vs. 10.6%; cannabis: 27.4% vs. 11.6%), and all sex differences in substance use reaching high levels of statistical significance (all *p* < 0.001).

### 3.2 Predictors of Lifetime Alcohol Use

Table 2 presents aPRRs and 95% CIs from the fully adjusted Poisson models for lifetime alcohol use. In the total sample, male sex (aPRR = 2.01; 95% CI: 1.39–2.90; *p* < 0.001), age older than 24 years (aPRR = 1.85; 95% CI: 1.14–3.01; *p* < 0.05), having relatives who used drugs during upbringing (aPRR = 1.41; 95% CI: 1.02–1.95; *p* < 0.05) and having friends who used drugs during upbringing (aPRR = 1.48; 95% CI: 1.04–2.10; *p* < 0.05) were each independently and positively associated with higher lifetime alcohol use prevalence. Conversely, Islamic religious affiliation (aPRR = 0.59; 95% CI: 0.41–0.84; *p* < 0.01) and residing at a friend's house (aPRR = 0.49; 95% CI: 0.25–0.97; *p* < 0.05) were inversely associated with alcohol use in the pooled model.

Among males specifically, those aged over 24 years had 3.5 times the prevalence of lifetime alcohol use compared to their younger peers (aPRR = 3.54; 95% CI: 1.62–7.74; *p* < 0.01), and those whose parents were together during upbringing had 2.7 times the prevalence of those from single‐parent or separated households (aPRR = 2.66; 95% CI: 1.51–4.70; *p* < 0.05). Furthermore, strict parental supervision (aPRR = 0.43; 95% CI: 0.22–0.83; *p* < 0.05) and the absence of family conflict (aPRR = 0.45; 95% CI: 0.24–0.83; *p* < 0.05) were each independently protective against alcohol use among males. Notably, Islamic faith, peer drug history and family drug history did not reach statistical significance in the male‐only model, suggesting that these total‐sample associations were partially driven by the female stratum.

Among females, Islamic religious affiliation was associated with significantly lower alcohol use prevalence (aPRR = 0.51; 95% CI: 0.28–0.93; *p* < 0.05), and having relatives who used drugs during childhood was associated with approximately twice the prevalence of alcohol use (aPRR = 1.97; 95% CI: 1.10–3.55; *p* < 0.05). An extreme near‐zero estimate was observed for females residing at a friend's house (aPRR ≈ 1.62 × 10^−7^). This arises from quasi‐complete separation—no female participant in this residence category reported lifetime alcohol use—and the estimate should not be substantively interpreted. It is retained for transparency.

### 3.3 Predictors of Lifetime Tobacco Use

Table 3 presents the fully adjusted Poisson model results for lifetime tobacco use. In the total sample, male sex was strongly associated with higher tobacco use prevalence (aPRR = 3.12; 95% CI: 2.10–4.64; *p* < 0.001). Additionally, affiliation with a non‐Christian, non‐Muslim religion (‘Other’ category) was independently associated with higher tobacco prevalence compared to Christianity (aPRR = 3.28; 95% CI: 1.37–7.82; *p* < 0.01). Conversely, the absence of family conflict (‘Never’ vs. ‘Sometimes’) was associated with lower tobacco use prevalence (aPRR = 0.59; 95% CI: 0.39–0.90; *p* < 0.05). No other variable reached statistical significance in the pooled model.

**TABLE 3:** Adjusted prevalence rate ratios (aPRR) and 95% confidence intervals (CI) for lifetime tobacco use, stratified by sex.

Variables	Total aPRR (95% CI)	Male aPRR (95% CI)	Female aPRR (95% CI)
Sex			
Female (ref)	1.00 (ref)	—	—
Male	3.12 (2.10–4.63)^***^	—	—
Age (years)			
≤24 (ref)	1.00 (ref)	1.00 (ref)	1.00 (ref)
>24	0.64 (0.34–1.19)	0.71 (0.30–1.67)	0.59 (0.20–1.71)
Religion			
Christianity (ref)	1.00 (ref)	1.00 (ref)	1.00 (ref)
Islam	1.41 (0.98–2.03)	2.19 (1.30–3.68)^**^	1.00 (0.58–1.72)
Other(s)	3.28 (1.37–7.83)^**^	3.81 (1.51–9.63)^**^	5.68 (0.83–38.73)
Marital status			
Married (ref)	1.00 (ref)	1.00 (ref)	1.00 (ref)
Single	0.80 (0.42–1.52)	0.59 (0.24–1.46)	0.97 (0.37–2.55)
Residence status			
Family house (ref)	1.00 (ref)	1.00 (ref)	1.00 (ref)
Renting/Alone	1.01 (0.68–1.49)	1.11 (0.63–1.96)	1.09 (0.60–1.99)
Friend's house	1.54 (0.60–3.97)	4.74 (1.24–18.03)^*^	1.31 (0.23–7.36)
Year of study			
Year 1 (ref)	1.00 (ref)	1.00 (ref)	1.00 (ref)
Year 2	1.04 (0.56–1.95)	1.02 (0.44–2.35)	1.25 (0.44–3.55)
Year 3	1.28 (0.70–2.32)	0.92 (0.40–2.14)	1.56 (0.55–4.41)
Year 4	1.24 (0.67–2.30)	0.73 (0.32–1.66)	1.98 (0.68–5.73)
Year 5	1.17 (0.57–2.41)	0.88 (0.37–2.11)	0.70 (0.14–3.49)
Year 6	1.45 (0.79–2.67)	0.67 (0.28–1.60)	1.98 (0.69–5.65)
Parents together (growing up)			
No (ref)	1.00 (ref)	1.00 (ref)	1.00 (ref)
Yes	1.52 (0.88–2.64)	1.53 (0.83–2.82)	2.01 (0.74–5.43)
Parental supervision			
Liberal (ref)	1.00 (ref)	1.00 (ref)	1.00 (ref)
Strict	0.77 (0.37–1.62)	0.87 (0.38–1.99)	0.61 (0.19–1.97)
Moderate	0.83 (0.40–1.72)	0.99 (0.42–2.32)	0.63 (0.18–2.16)
Family quarrels			
Sometimes (ref)	1.00 (ref)	1.00 (ref)	1.00 (ref)
Always	0.87 (0.60–1.27)	1.25 (0.72–2.16)	0.55 (0.24–1.28)
Never	0.59 (0.39–0.90)^*^	0.83 (0.48–1.43)	0.44 (0.16–1.20)
Relatives using drugs (growing up)			
No (ref)	1.00 (ref)	1.00 (ref)	1.00 (ref)
Yes	1.18 (0.82–1.70)	1.19 (0.75–1.88)	1.05 (0.56–1.97)
Friends using drugs (growing up)			
No (ref)	1.00 (ref)	1.00 (ref)	1.00 (ref)
Yes	1.20 (0.83–1.73)	0.56 (0.33–0.96)^*^	2.49 (1.35–4.58)^**^
Lifetime alcohol use			
No (ref)	1.00 (ref)	1.00 (ref)	1.00 (ref)
Yes	0.87 (0.56–1.36)	0.85 (0.47–1.53)	0.93 (0.42–2.06)
Lifetime cannabis use			
No (ref)	1.00 (ref)	1.00 (ref)	1.00 (ref)
Yes	0.96 (0.63–1.46)	1.03 (0.61–1.74)	0.96 (0.41–2.23)

*Note:* All models simultaneously adjusted for all listed variables.

Abbreviations: aPRR, adjusted prevalence rate ratio; CI, confidence interval; ref, reference category.

^***^ *p* < 0.001.

^**^ *p* < 0.01.

^*^ *p* < 0.05.

Among males, those who identified with non‐Christian religions were more likely to report lifetime tobacco use, including both Islamic affiliation (aPRR = 2.19; 95% CI: 1.30–3.68; *p* < 0.01) and other religions (aPRR = 3.81; 95% CI: 1.51–9.62; *p* < 0.01) relative to Christians. Furthermore, males residing at a friend's house had 4.7 times the tobacco use prevalence of those in a family home (aPRR = 4.74; 95% CI: 1.25–18.03; *p* < 0.05), consistent with a reduced supervision and heightened social exposure effect. Notably, among males, having friends who used drugs during upbringing was inversely associated with tobacco use (aPRR = 0.57; 95% CI: 0.33–0.97; *p* < 0.05). This counterintuitive inverse association may reflect multicollinearity between peer drug history and other predictors in the male‐only model and should be interpreted with caution.

Among females, having friends who used drugs during upbringing was the only statistically significant predictor of lifetime tobacco use, with approximately 2.5 times the prevalence compared to those without such peer exposure (aPRR = 2.49; 95% CI: 1.35–4.58; *p* < 0.01). No other variable was significantly associated with tobacco use in the female‐only model. The limited number of female tobacco users (*n* = 43) likely reduced statistical power to detect associations of moderate effect size in the female stratum.

### 3.4 Predictors of Lifetime Cannabis Use

Table 4 presents aPRRs and 95% CIs from the adjusted Poisson models for lifetime cannabis use. In the pooled total‐sample model, male sex was the sole statistically significant predictor, with males having approximately 2.6 times the prevalence of lifetime cannabis use compared to females (aPRR = 2.56; 95% CI: 1.71–3.84; *p* < 0.001). No other variable was independently associated with cannabis use in the pooled model after adjustment.

**TABLE 4:** Adjusted prevalence rate ratios (aPRR) and 95% confidence intervals (CI) for lifetime cannabis use, stratified by sex.

Variables	Total aPRR (95% CI)	Male aPRR (95% CI)	Female aPRR (95% CI)
Sex			
Female (ref)	1.00 (ref)	—	—
Male	2.56 (1.71–3.84)^***^	—	—
Age (years)			
≤24 (ref)	1.00 (ref)	1.00 (ref)	1.00 (ref)
>24	1.46 (0.80–2.68)	1.81 (0.72–4.55)	1.65 (0.64–4.25)
Religion			
Christianity (ref)	1.00 (ref)	1.00 (ref)	1.00 (ref)
Islam	0.89 (0.63–1.24)	0.54 (0.33–0.90)^*^	1.47 (0.87–2.49)
Other(s)	0.78 (0.30–2.04)	0.45 (0.15–1.34)	3.70 (0.51–26.94)
Marital status			
Married (ref)	1.00 (ref)	1.00 (ref)	1.00 (ref)
Single	0.91 (0.48–1.72)	0.82 (0.36–1.88)	1.02 (0.37–2.84)
Residence status			
Family house (ref)	1.00 (ref)	1.00 (ref)	1.00 (ref)
Renting/Alone	1.55 (0.98–2.44)	1.04 (0.54–2.02)	3.48 (1.65–7.35)^**^
Friend's house	1.65 (0.71–3.85)	1.42 (0.40–5.03)	2.86 (0.66–12.39)
Year of study			
Year 1 (ref)	1.00 (ref)	1.00 (ref)	1.00 (ref)
Year 2	1.38 (0.73–2.61)	0.87 (0.35–2.14)	1.52 (0.58–3.98)
Year 3	1.62 (0.87–3.00)	1.25 (0.54–2.87)	1.34 (0.50–3.60)
Year 4	0.90 (0.45–1.78)	0.27 (0.08–0.86)^*^	1.54 (0.57–4.18)
Year 5	1.67 (0.82–3.39)	1.25 (0.49–3.23)	1.29 (0.41–4.10)
Year 6	1.31 (0.70–2.47)	0.96 (0.41–2.26)	1.72 (0.64–4.62)
Parents together (growing up)			
No (ref)	1.00 (ref)	1.00 (ref)	1.00 (ref)
Yes	1.06 (0.67–1.67)	0.96 (0.48–1.91)	0.88 (0.42–1.83)
Parental supervision			
Liberal (ref)	1.00 (ref)	1.00 (ref)	1.00 (ref)
Strict	0.75 (0.40–1.41)	0.67 (0.31–1.43)	0.81 (0.30–2.18)
Moderate	0.82 (0.45–1.48)	0.68 (0.33–1.40)	1.01 (0.37–2.75)
Family quarrels			
Sometimes (ref)	1.00 (ref)	1.00 (ref)	1.00 (ref)
Always	1.33 (0.84–2.09)	0.88 (0.46–1.71)	2.56 (1.20–5.46)^*^
Never	0.71 (0.43–1.18)	0.78 (0.40–1.50)	0.68 (0.29–1.59)
Relatives using drugs (growing up)			
No (ref)	1.00 (ref)	1.00 (ref)	1.00 (ref)
Yes	1.25 (0.88–1.77)	1.21 (0.74–1.97)	1.32 (0.72–2.40)
Friends using drugs (growing up)			
No (ref)	1.00 (ref)	1.00 (ref)	1.00 (ref)
Yes	1.35 (0.94–1.92)	1.22 (0.73–2.02)	1.62 (0.87–3.02)

*Note:* All models simultaneously adjusted for all listed variables. Column order: male, then female (consistent with Tables 2 and 3).

Abbreviations: aPRR, adjusted prevalence rate ratio; CI, confidence interval; ref, reference category.

^***^ *p* < 0.001.

^**^ *p* < 0.01.

^*^ *p* < 0.05.

Among males, Islamic religious affiliation was associated with significantly lower cannabis use prevalence compared to Christianity (aPRR = 0.54; 95% CI: 0.33–0.90; *p* < 0.05), and being in the fourth year of study was associated with lower cannabis use prevalence compared to first‐year students (aPRR = 0.27; 95% CI: 0.08–0.87; *p* < 0.05). No other predictors reached statistical significance in the male‐only model.

Among females, two factors were significantly and independently associated with cannabis use. First, renting or living independently was associated with 3.5 times the prevalence of cannabis use compared to living in a family home (aPRR = 3.48; 95% CI: 1.65–7.35; *p* < 0.01). Second, persistent family conflict (‘always’ vs. ‘sometimes’) was associated with approximately 2.6 times the cannabis use prevalence (aPRR = 2.56; 95% CI: 1.20–5.46; *p* < 0.05).

## 4 Discussion

### 4.1 Overview of Key Findings

This study examined sex differences in the factors associated with lifetime alcohol, tobacco and cannabis use among undergraduate students at the UDS, Ghana. Overall, lifetime prevalence was 17.7% for alcohol, 17.0% for tobacco and 16.7% for cannabis, with males reporting substantially higher use across all three substances than females. Sex‐stratified adjusted modified Poisson regression analyses revealed distinct risk and protective factor profiles for males and females—associations that, in several important instances, were either obscured or absent in the pooled total‐sample model. These findings affirm the critical importance of sex‐stratified analyses in substance use research.

### 4.2 Prevalence of Lifetime Substance Use and Sex Differentials

The observed lifetime prevalence rates—17.7% for alcohol, 17.0% for tobacco and 16.7% for cannabis—are markedly lower than those reported in comparable studies among Ghanaian tertiary students (e.g., 54.8% for alcohol [7]) and elsewhere in sub‐Saharan Africa [2, 3]. This pattern is likely attributable to the specific socioreligious context of UDS and Tamale: with approximately half the student population identifying as Muslim—for whom alcohol and tobacco are explicitly prohibited—and in a predominantly conservative Northern Ghanaian city with strong communal and religious oversight, substance use initiation is substantially moderated by social norms. Nevertheless, the sex‐stratified rates among males are more pronounced, with 31.1% reporting lifetime alcohol use, 30.6% tobacco use and 27.4% cannabis use, figures more closely aligned with rates reported among male university students in Ethiopia [4] and Nigeria [24].

It is important to note that the figure of 31.1% represents the within‐sex prevalence of alcohol use among male participants (i.e., the proportion of male participants who reported lifetime alcohol use). It does not represent the proportion of all alcohol users who were male, which was 56.6% (60 of 106 users). These are conceptually distinct metrics, and conflating them would mischaracterise the direction and magnitude of sex disparities. The sex differentials observed here are consistent with global literature showing that males consume alcohol and tobacco at substantially higher rates than females [28, 29, 30]. These disparities are frequently attributed to differential social permissions—in many Ghanaian and West African contexts, substance use is more culturally condoned among men—and to greater male exposure to peer drinking environments [31, 32].

### 4.3 Sex‐Specific Predictors: Alcohol Use

Male sex was the strongest predictor of lifetime alcohol use in the total sample, confirming that being male approximately doubles the prevalence of lifetime alcohol use, independent of all other measured factors. This is consistent with evidence from Uganda [33], Nigeria [24] and broader West African reviews [34, 35]. Furthermore, older age (>24 years) predicted higher alcohol use in the total sample and, even more strongly, among males specifically, consistent with the trajectory of social independence, income access and reduced oversight that accompanies advancing student years [35, 36].

A notable and counterintuitive finding among males was that having parents together during upbringing was associated with higher alcohol use prevalence. This association has been documented previously and likely reflects permissive or inconsistent parenting styles within intact families, rather than family structure per se [37]. As Mathibela and Skhosana [37] explain, parenting quality—characterised by monitoring, rule‐setting and emotional engagement—is a more proximal determinant of adolescent risk behaviour than household composition alone. This finding points to the importance of parenting quality interventions in intact families. Additionally, it may be that the high percent of females with strict parental supervision (∼60%) precludes any impact on alcohol use. Males were less strictly supervised; therefore, there can be a significant difference between those who do and do not use alcohol. Among females, it may be that strict parental supervision is so common that it does not differ between those who do and do not use alcohol.

Conversely, strict parental supervision was independently protective among males, but no equivalent significant effect was found among females. This sex‐specific pattern suggests that external behavioural monitoring is particularly salient in curtailing male alcohol initiation, consistent with evidence that male substance use is more responsive to environmental constraints during formative years [38]. Similarly, the absence of family conflict was protective against alcohol use among males, but not among females. This sex difference may reflect that family conflict activates distinct coping pathways by sex: males may be more likely to escape familial tension through substance use, whereas females may employ alternative coping strategies such as social support‐seeking or religious engagement [7].

Among females, Islamic faith was independently protective against alcohol use but did not reach significance in males. This finding may reflect that religious prohibitions are more tightly enforced within female social networks in Northern Ghana, or that the smaller male stratum reduced the power to detect an effect of similar magnitude. Family modelling of drug use was a significant risk factor among females but not males—a sex‐specific pathway consistent with evidence that familial socialisation exerts particularly strong influences on female substance use initiation through modelling and normalisation processes [13, 39].

### 4.4 Sex‐Specific Predictors: Tobacco Use

Male sex was an exceptionally strong predictor of lifetime tobacco use in the total sample, reflecting the markedly higher cultural tolerance and social exposure to tobacco among males in Ghana and across sub‐Saharan Africa [31, 40]. Notably, in the total sample, affiliation with non‐Christian, non‐Muslim religions (‘Other’ category) was associated with approximately 3.3 times the tobacco prevalence of Christians. This small category (*n* = 9) likely includes indigenous African religious practitioners, for whom tobacco may carry ritual significance; however, the small cell sizes and wide CI suggest this finding should be replicated before strong conclusions are drawn.

Among males, the positive associations between Islamic affiliation and tobacco use and residence at a friend's house and tobacco use are striking. The Islamic faith finding is paradoxical given that Islam prohibits tobacco use; however, it has been documented in several prior studies that the association between Islamic faith and reduced substance use is complex and context‐dependent, particularly in settings where Islamic prohibition may be inconsistently observed [40, 41]. The wide CI further indicates imprecision in this estimate. The friend's house finding, by contrast, is intuitively consistent with the idea that males in shared accommodation without family supervision are substantially more exposed to social environments conducive to tobacco experimentation.

Among females, peer drug exposure during upbringing was the sole significant predictor of lifetime tobacco use. This pattern stands in stark contrast to males, where peer drug history was inversely (and counterintuitively) associated with tobacco use. The positive association among females is coherent with social learning theory: female students whose early social environments normalised drug use through peer behaviour are more likely to initiate tobacco use, an effect that may be amplified by the relatively fewer alternative social influences that female students from drug‐using peer environments encounter in a culturally conservative university setting [42]. This finding explicitly identifies peer socialisation as a female‐specific tobacco risk pathway—one that would be attenuated or masked in any pooled analysis.

### 4.5 Sex‐Specific Predictors: Cannabis Use

For cannabis use, male sex remained the dominant total‐sample predictor, consistent with global and regional patterns attributing higher cannabis use to males through pathways of social exposure, peer normalisation and risk tolerance [43, 44, 45]. The absence of other significant total‐sample predictors after full adjustment suggests that, once sex and the shared confounders are accounted for, sociodemographic and family factors explain limited additional variance in cannabis use at the population level.

However, sex‐stratified analysis uncovers a richer picture. Among males, Islamic faith was protective, consistent with the religious prohibition of all intoxicants in Islam [41, 46] and with the finding for alcohol above. Among males in the fourth year of study, cannabis use was substantially lower than among first‐year students. This may reflect a selection effect. For instance, students who initiated and continued cannabis use may have been more likely to discontinue their studies before reaching year four, or it may reflect a genuine developmental shift towards reduced recreational risk‐taking as academic engagement and career investment deepen in later years.

Among females, two factors emerged as sex‐specific risk factors. Residing independently (renting or living alone) was associated with 3.5 times the cannabis use prevalence compared to living in a family home. Moreover, persistent family conflict was associated with 2.6 times the prevalence relative to occasional conflict. Neither association was significant in males or in the pooled model. These findings suggest that for female students, residential autonomy and household distress may operate as compounding pathways to cannabis initiation: residential independence reduces protective supervision, whereas family conflict may generate emotional distress that motivates substance use as a coping mechanism [8, 47, 48]. This psychosocial stress‐coping pathway, particularly relevant for females, is consistent with broader literature on gender and drug use motivation [28, 29].

### 4.6 Cross‐Substance Considerations

Across the three outcome models for tobacco and cannabis, prior or co‐occurring use of the other substances was included as an adjustment variable. In none of the models did lifetime use of one substance emerge as a statistically significant predictor of another after adjustment for the full suite of sociodemographic and psychosocial covariates. This does not indicate an absence of polydrug use in the sample; rather, in fully adjusted simultaneous models, the shared sociodemographic predictors of substance use may account for much of the correlation between substances, leaving limited residual variance attributable to cross‐substance use. Additionally, the ‘ever‐use’ operationalisation may obscure temporally ordered patterns of co‐use that could only be captured longitudinally. Future research should employ dedicated polydrug use frameworks, such as latent class analysis or network analysis to characterise polydrug patterns and their determinants more precisely.

### 4.7 Strengths and Limitations

This study has several notable strengths. First, the sex‐stratified analytical approach, using adjusted modified Poisson regression with robust standard errors, enabled precise estimation of sex‐specific predictors while appropriately controlling for potential confounders. This approach provides added analytical rigour beyond simple sex‐disaggregated descriptive reporting. Second, the study was based on a systematically sampled cross‐institutional sample within UDS, with a 100% response rate, minimising non‐response bias. Third, the use of an internationally validated base instrument (the WHO Student Drug Use Survey) ensured comparability with prior literature.

Nevertheless, several limitations must be acknowledged. The cross‐sectional design precludes causal inference; all associations are cross‐sectional and may reflect shared antecedents rather than directional relationships. Further, the lifetime substance use measure (ever‐use at least once) cannot distinguish single‐instance experimentation from regular or dependent use, potentially conflating meaningfully different patterns of substance involvement. Again, self‐reported substance use in a culturally conservative, primarily Muslim Northern Ghanaian setting is likely subject to social desirability bias; despite anonymisation, some under‐reporting is plausible, which may have suppressed estimated prevalences. Nonetheless, the formal psychometric validation of the adapted instrument in the specific UDS student population was not conducted; future studies should assess measurement reliability and validity in this context. However, the sex distribution of the sample (67.8% female, 32.2% male) reflects the actual UDS student sex composition but resulted in a smaller male stratum (*n* = 193), reducing statistical power in male‐specific models and potentially leaving associations of moderate effect size undetected among males. Additionally, the quasi‐complete separation phenomenon in the female alcohol model (for the friend's house residence category) highlights a fundamental limitation of maximum likelihood Poisson estimation in very small sub‐groups; Bayesian estimation with regularising priors could address this in future work. Moreover, the sample encompasses a single university in Northern Ghana, limiting generalisation to other Ghanaian universities, particularly those in southern or coastal Ghana, where the socioreligious context and substance use norms differ substantially. Finally, unmeasured confounders, including campus culture, academic workload, mental health status, financial stress and neighbourhood substance availability, may have biased the associations observed.

### 4.8 Implications

Notwithstanding these limitations, the findings carry important and actionable public health implications. The consistently higher substance use prevalence among males, combined with the identification of sex‐specific risk factors, argues strongly for sex‐differentiated prevention strategies rather than uniform campus‐wide programmes. For male students, programmes strengthening parental engagement and monitoring, fostering family harmony and providing structured social alternatives during periods of academic transition (particularly for older and later‐year students) are likely to be most impactful. For female students, interventions addressing the psychosocial risks of residential independence—including mentoring, peer support groups and accessible counselling services within university residences—and the emotional consequences of family conflict may reduce cannabis initiation. Furthermore, the consistently protective role of religious affiliation, particularly Islam, across multiple substances and both sexes supports the strategic involvement of faith‐based community leaders and religious institutions in campus prevention partnerships. Future research should prioritise longitudinal designs, validated psychometric instruments and polydrug use frameworks to deepen the evidence base for sex‐sensitive substance use prevention in Ghanaian higher education institutions and comparable settings across sub‐Saharan Africa.

## 5 Conclusions

This study demonstrates that sex differences profoundly and differentially shape the prevalence and predictors of lifetime alcohol, tobacco and cannabis use among undergraduate students at the UDS, Ghana. Males consistently report higher lifetime use across all three substances. However, the determinants of substance use are not simply the mirror image of each other across sexes; they are qualitatively distinct in important ways. Among males, strict parental supervision, the absence of family conflict and Islamic faith are key protective factors, whereas older age and peer or family drug history drive risk. Among females, Islamic faith protects against alcohol use, whereas residential autonomy and persistent family conflict uniquely elevate cannabis risk—pathways that are entirely obscured in pooled analyses. Sex‐stratified adjusted modified Poisson regression analysis is therefore indispensable for generating the sex‐specific evidence needed to guide targeted prevention. Taken together, these findings underscore the urgent need for sex‐sensitive, culturally grounded and institutionally supported substance use prevention strategies within Ghanaian higher education. Future longitudinal and qualitative research is needed to illuminate the causal mechanisms linking sex, social context and substance use in this population.

## Author Contributions

Yula Salifu and Joseph Lasong conceived and designed the study. Yula Salifu, Joseph Lasong, Amidu Alhassan, Frank Offei Odonkor and Torjim Salifu collected and managed data. Yula Salifu and Joseph Lasong conducted statistical analyses and interpreted results. Yula Salifu, Joseph Lasong, Amidu Alhassan, Frank Offei Odonkor and Torjim Salifu drafted and revised the manuscript. All authors read and approved the final version.

## Funding

The authors have nothing to report.

## Ethics Statement

Ethical approval was granted by the IRB of the University for Development Studies. Administrative approval was obtained from Deans and Heads of Department of participating schools. The study complied with the Declaration of Helsinki (2013 revision). Responses were anonymised and coded to protect participant confidentiality; no names or index numbers were retained in the analytic dataset. The study was reported in accordance with the Strengthening the Reporting of Observational Studies in Epidemiology (STROBE) guidelines for cross‐sectional studies [27].

## Consent

Written informed consent was obtained from participants aged ≥18 years; participants below 18 years provided written assent alongside parental/guardian written informed consent.

## Conflicts of Interest

The authors declare no conflicts of interest.

## Data Availability

The datasets used and/or analysed during the current study are available from the corresponding author on reasonable request.
